# HIV and mental health: An overview of research from India

**DOI:** 10.4103/0019-5545.69245

**Published:** 2010-01

**Authors:** Nishanth Jayarajan, Prabha S. Chandra

**Affiliations:** Junior Resident, Department of Psychiatry, NIMHANS, Bangalore, India; 1 Professor of Psychiatry, Department of Psychiatry, NIMHANS, Bangalore, India

**Keywords:** AIDS, HIV, India, mental health

## Abstract

HIV/AIDS has gained prominence in India as a growing public health issue. There is a complex but significant interaction between mental health and HIV/AIDS. HIV affects mental health by its direct neurobiological action, the impact of having the illness, by its treatment including that for opportunistic infections and by its impact on the family. In addition, substance use and mental illness as vulnerability factors add to the complexity of assessment, differential diagnosis and management. This paper reviews literature published in India on the topic.

## INTRODUCTION

Since its emergence in the 1980s, HIV has been an enigma across medical specialties. Despite early calls for preventive administrative measures voiced by different parts of the medical community, including the psychiatric fraternity,[1] the disease has spread and India has several pockets of epidemics in different parts of the country.[2] Currently, India has an estimated prevalence of 0.23-0.33%.[3] Research from developing countries into this area has been exceedingly sparse, bearing in mind the fact that the epidemic has disproportionately affected the southern hemisphere. Public health efforts have been lethargic in tackling the double danger of HIV and mental illness.

Assessment and management of mental disorders is integral to an effective HIV/AIDS intervention program. Mental health professionals will increasingly be called upon to assist in the management of people living with HIV/AIDS. Thus psychiatrists will need to be familiar with disorders that are prevalent in HIV infection and also the interface of treatment, including HAART with mental health. This article is an attempt to throw light on these issues from an Indian perspective, by putting together the available data from Indian studies in this regard.

## BIDIRECTIONAL LINK OF HIV AND MENTAL ILLNESS

The relation between HIV and mental illness has been studied by examining HIV infection in those with mental illness and mental illness in those with HIV. However, there are many common factors in both, such as homelessness, incarceration, poverty and substance misuse. There is some evidence to suggest that HIV risk in people with severe mental illness is mediated through substance misuse.[4] In addition to this avenue of investigation, there has been exploration of the impact of psychological morbidity on disease progression, response to treatment and outcome of treatment.

## HOW ARE THE MENTALLY ILL AT MORE RISK?

There is increasing evidence of prevalence of HIV and high-risk behavior among psychiatric patients. There is a significant body of research from India [Table 1] examining the link between HIV and mental health. Evidence from developing countries is more limited[5] with four studies from South Asia.[6–9] HIV prevalence of 1.7% has been reported among psychiatric inpatients.[9] The predominant risk behavior among psychiatric patients in India is unprotected heterosexual intercourse, which reflects the common mode of transmission in the country.[6,10–12] Prevalence of risk behavior ranges from a lifetime history in 26% (men) and 11% (women) and recent history in 5% men and 6% women[10–12] although previously much higher rate of 51% has been reported in inpatients.[7] Patients with comorbid substance misuse are more likely to engage in HIV risk behavior and lack of adequate knowledge about HIV also contributes to it.[7] Women with severe mental illness have a higher prevalence of high-risk behavior in those with a history of abuse.[10–12]

**Table 1:** Psychological morbidity in HIV

Jacob *et al*. (1991)	Psychiatric morbidity in HIV	N = 46 Major depression and adjustment disorder commonest diagnoses after revealing seropositivity
Ahuja *et al*.[32]	Psychiatric morbidity in HIV	Higher prevalence of psychiatric disorders as compared to the general population
Chandra *et al*.[6]	Psychological morbidity in HIV infection	40% Depression
		36% Anxiety
		14% Suicidal ideas
Joseph and Bhatti[44]	Psychosocial problems in HIV N = 30 HIV positive women	Escape avoidance the most preferred coping strategy adopted
Yepthomi *et al*.[18]	30 advanced HIV vs. control Cognitive battery	Cognitive difficulties prevalent
		56% of the patients with advanced HIV meeting the criterion for impairment in two cognitive domains
Rao *et al*.[34] (unpublished data cited)	Psychiatric morbidity in HIV infected children (determined by K SADS-PL)	45% had a lifetime prevalence of any psychiatric illness
		Anxiety and behavioral disorders were the common psychiatric illness as a group (18% each)
Grover *et al*.[41]	Behavioral disorder in HIV infected children	Significantly higher prevalence of behavioral disorders in HIV infected children compared to controls. HIV infection and disturbed family environment most consistent correlates of behavioral disorder
Mittal *et al*. (2007)	Psychiatric morbidity in AIDS patients	5% of the patients were suffering from depressive disorders, 12% had GAD, 10% had drug dependence, 3% had panic disorder, 2% had schizophrenia and 2% had personality disorders.
Ramasubramanian *et al*.[33]	PTSD scores in HIV/AIDS vs. control	PLWHA had more and significant PTSD scores than their counterparts
Mandal[51]	Neurocognitive impairment in HIV Case control 50:50	Seropositive patients had poorly performed in digit symbol substitution test, trail making test and controlled word association test. Not related to duration of illness

## WHAT ARE THE PSYCHIATRIC COMORBIDITIES IN HIV?

Psychiatric comorbidity in HIV ranges from minor cognitive deficits to frank psychosis. Since the early 1990s there have been efforts to document the neuropsychiatric aspects of HIV.[13] Psychiatric manifestations are more in HIV-affected individuals as compared to other STDs.[14] There is considerable evidence that depression and anxiety are prevalent diagnoses among those with HIV infection.[15–16] Cognitive deficits in HIV vary from subtle abnormalities in attention and concentration through to gross psychomotor retardation and dementia. It is well established that HIV associated dementia involves most cognitive domains, but evidence on early changes are less consistent[17] [Table 1].

### Cognitive deficits

In India, significant cognitive deficits are reported in advanced HIV disease in patients not receiving HAART. In one study, 56% of PLWHA were demonstrated to have impairment in at least two cognitive domains.[18] Neurocognitive disturbances in asymptomatic HIV infection have been a subject of research interest in view of the implications on its influence on occupational functioning. Between 60-90% of asymptomatic subjects with HIV have been reported to have cognitive deficits.[19,20] Specific deficits have been reported in digit symbol substitution test, trail making test and controlled word association test.[21] The duration of detected illness does not appear to have a significant relation to the degree of deficits. There have been reports of neurocognitive impairment increasing with worsening clinical status.[22] A follow-up study of neuropsychological function at baseline with six-monthly reassessments found that only one of 10 cognitive variables-visual working memory showed deterioration over 30 months.[23]

There has been extensive research into differences in neuropathology between different clades of HIV using animal studies and human fetal cells. HIV 1 clade C, the prevalent type in India has been found to have less toxic form of viral protein as compared to the clade B.[24,25] Mishra *et al*.[25] suggest this as a possible reason for the difference in degree of HIV-1 associated neurological deficits in India. Delirium is common in HIV and those with advanced AIDS and dementia are particularly vulnerable. Diarrhea, hypoxia related to pneumocystis carini infection, neuro infections, alcohol withdrawal and some drugs used in treatment could all contribute. In the Indian context, one should also enquire for use of alternate forms of treatment or traditional medicines that may contribute to delirium.

### Psychosis

Psychotic symptoms seen in HIV-infected individuals may be primary or secondary.[26] Occasionally psychotic symptoms may be the presenting complaints of an HIV illness.[27] One case report highlights the presentation of progressive multifocal leukoencephalopathy (PML) being camouflaged by catatonic symptoms, thereby emphasizing the need for detailed investigations in such a presentation.[28] Primary psychosis does not yield any signs of HIV cerebral disease whereas secondary psychosis often occurs in the context of global (encephalopathy) or localized pathology (most often lesions of the left temporal lobe and diencephalon). Other factors that need to be considered in the differential diagnosis include presence of opportunistic infections like tuberculoma, toxoplasmosis and cryptococcal meningitis, which may present as acute psychosis in the initial stages.

Drugs like INH can also contribute to psychosis and co occurrence of neurosyphilis may also lead to psychosis. Several patients with comorbid substance use may present with withdrawal related psychosis following a sudden infection or hospitalization.

### Depression

Emotional problems are among the most common symptoms in HIV patients with up to 98.6% prevalence.[29] Depression is a prevalent comorbidity in HIV infection as well as a recognized side-effect of NRTI, Protease inhibitors and NNRTIs. It may also be the first presenting symptom in an HIV case.[30] It is essential to discriminate between normal response to a life threatening illness, clinical manifestation of HIV and depressive episode while recognizing that all three can coexist. As in other serious medical illness, anhedonia may be the most reliable indicator of severe depression. HIV infected individuals are recognized to be at high risk of suicide in the period immediately after coming to know of seropositive status, especially if they have a past psychiatric history.[4] Chronic pain, commonly encountered in HIV, both due to disease as well as treatment related side-effects, is often associated with depression.

### Mania

Mania is overrepresented in HIV infection compared to general population. A case series explored the various possible associations of HIV and mania like manic symptoms being a direct effect of the illness, effect of HAART drugs, or as a reaction to disclosure of the diagnosis.[31] Although manic episodes can occur early in the infection, it is more common in later phases of the infection, often associated with cognitive deficits and can be a presentation of HIV dementia or associated with psychosis.

### Anxiety disorders

Among those with HIV, up to 28% may have adjustment disorder,[32] 25-36% may suffer from anxiety[4,11] and there is a higher prevalence of PTSD scores among people living with HIV/AIDS.[33] Anxiety is also prevalent among children with HIV (18%).[34]

A planned wedding or sex between the couple, in the context of being diagnosed with HIV, can precipitate and maintain anxiety disorders. Drugs, both prescribed and illicit should be considered in the etiology but most commonly in this population, alcohol misuse can maintain the disorder resulting in poor response to treatment. Psychiatric assessment should aim at identifying specific precipitating factors for anxiety disorder.

### Suicide

HIV can be a significant risk factor for suicide. Chronic pain, anxiety and depression should prompt a thorough suicidal risk assessment. Suicidal attempt is most likely to occur in those with a history of psychiatric illness and in the immediate aftermath of diagnosis with HIV.[4]

### Bereavement

Complicated grief reactions among relatives are common sequelae of death due to HIV infection. Up to 40% of HIV infected children have been reported to be orphaned.[35] HIV deaths may often be stigmatized leading to a lack of funeral rituals, which are an important part of societal mourning in this region.[36] Psychological intervention is appropriate if grief is unresolved in the context of dissipating and often hostile social support system.[37]

## HOW DO PSYCHOLOGICAL FACTORS AFFECT PROGNOSIS IN HIV?

### Psychoneuroimmunology

Chittiprol *et al*.[20] followed up a sample of 120 HIV seropositive (including HIC 1 C), neurologically asymptomatic subjects to investigate endocrine functions. They attribute the finding of consistently high cortisol response but inconsistent ACTH response to challenge in seropositive to HIV-1C infection adversely affecting the adaptability of the HPA axis to the stressor/s. They also found that the poor response of autonomic system in HIV positive subjects (as measured by epinephrine and norepinephrine levels following cognitive challenge) was consistent over time. A correlation between QOL scores on the physical health domain of QOL and CD4 counts was reported by Kohli *et al*.[38] Chandra *et al*.[7] found low CD4 counts (<200/mm) to be associated with low scores on the psychological and social relationships domain.

### Adherence to treatment

Psychiatric illness can be an important factor determining the adherence to treatment of HIV infection. Those with mental illness can have difficulty in adhering to the medication routine. Negative attitudes from health professionals may lead the patient to disengage from treatment.[39] In a sample of 310 patients on HAART, Sarna *et al*.[40] found that patients with severe depression were four times more likely to report lower adherence to treatment.

### Childhood psychiatric disorder in HIV

Children with HIV infection often grow up in stressful environment. Most acquire the disease through maternal transmission and are faced with parents living with a chronic life threatening condition. Parents’ death in such circumstances can leave the child stigmatized and with fragmented social support. Grover *et al*.[41] studied behavioral disorder by comparing 140 HIV positive children with age and family income matched HIV negative controls using Child Behavior Checklist. 19.3% of HIV infected children scored within the normal range on CBC in contrast to 81.7% of controls. The authors found that HIV infection and disturbed family environment were the most consistent correlates of behavioral disorder. There has been one unpublished data cited by Rao *et al*.[34] In 22 HIV infected pediatric outpatients, a prevalence of 45% psychiatric disorders (using K-SADS-PL) and 40% prevalence of behavioral problems (Child Behavior Checklist) are reported.

## WHAT ARE THE PSYCHOSOCIAL ASPECTS OF LIVING WITH HIV/AIDS?

The earliest psychological impact of being diagnosed with HIV can be understood within the framework of Kübler-Ross cycle of grief involving denial, anger, bargaining, depression and acceptance. However, the most important additional aspect in HIV/AIDS is the social stigma. Soon after becoming aware of one’s seropositive status, the HIV infected patient often has to work through life changes including relationships, family, employment, finances etc. Disclosure of seropositivity can be a stressful decision. If the individual feels the need to disclose and the outcome of disclosure is positive, this can be associated with better quality of life.[11] Quality of life in the early asymptomatic stage of illness is usually better than early symptomatic or AIDS stage with impact on both physical and psychological domains. Quality of life can be influenced by educational status and income as well.[42] When symptomatic a range of factors such as physical health, employment and social and biological function can impact upon quality of life.[38] Tarakeshwar *et al*.[43] studied 50 adults with HIV with regards to their beliefs that helped manage the illness and found that all 50 believed God to be a benevolent force. The spiritual practices were described as enabling them to face their troubles with less fear and greater confidence. In most low and middle-income countries only a minority of the population have access to HAART, and a significant proportion of patients end up without active treatment.

The key issues at the interface of psychiatry and palliative care for HIV are predominantly related to disorders such as delirium, dementia, substance dependence or withdrawal and depression. Psychiatrists may also be called upon to assess competence of the patient to make end of life decisions, offer advice on difficult family dynamics as well as staff stress and burnout.

### Women with HIV

Joseph and Bhatti[44] studied psychosocial problems in 30 HIV positive women and among other difficulties found compromised help seeking as a consequence of stigma with gender discriminatory and inadequate care. Gupta *et al*.,[45] found that HIV-positive women were significantly more likely to report marital dissatisfaction, a history of forced sex, domestic violence, depressive symptoms and husband’s extra marital sex when compared to HIV-negative women. Kohli *et al*.[38] also reported on significantly lower QOL scores in women despite having less advanced disease. Another study comparing quality of life in men and women with HIV found that men reported better quality of life in the environmental domain and women had higher scores on the spirituality/religion and personal beliefs domain.[46]

### Injectable drug users

The prevalence of HIV among IDUs has increased over time.[47] There is evidence for participation by women IDU in HIV prevention programs, though the effect was seen more in terms of practicing safer sex than safer injections.[48]

### Mental health Issues among MSM with HIV Infection

Men who have sex with men are at high risk for HIV and this group has been poorly studied in India compared to the Western world. This group includes Kothis (receptive, feminine), Panthis (penetrative, masculinized) and Hijras (transgender, hermaphrodite, castrated). The Kothis and Hijras are more likely to bear the brunt of social stigma with HIV as they are bracketed with female commercial sex workers and have less support coming their way.[49] Pandya[50] has described the range of psychosocial stressors that are faced across the lifespan by MSM in India. It is crucial that psychiatrists sensitively inquire regarding sexual preferences even when the patient is in a heterosexual relationship.

## CONCLUSIONS

From the available data, the intertwined relationship of HIV and psychiatric disorders is clear. Mental health professionals need to be aware of the varied psychiatric manifestations of HIV, and the impact of HIV on a pre-existing psychiatric condition. In addition, other issues like impact of opportunistic infections on the brain, the impact of illness on the life of the individual, the association of substance use and HIV, and the relationship of treatment and mental health need to be addressed. HIV challenges the psychiatrist to consider systematic and diverse methods in assessment, consider several possibilities in the differential diagnosis and also be aware of the problems related to use of different medications. In developing countries like India, specific issues such as comorbid infections, IV drug use and stigma and inadequate facilities for HAART and palliative care add to the mental health burden.
